# Identification of cuproptosis-related subtypes, characterization of tumor microenvironment infiltration, and development of a prognosis model in breast cancer

**DOI:** 10.3389/fimmu.2022.996836

**Published:** 2022-08-19

**Authors:** Zhi Li, Hua Zhang, Xixi Wang, Qun Wang, Jiapeng Xue, Yun Shi, Minghua Wang, Geng Wang, Jianquan Zhang

**Affiliations:** ^1^ Department of General Surgery, Taihe Hospital, Hubei University of Medicine, Shiyan, China; ^2^ Hubei Key Laboratory of Embryonic Stem Cell Research, Taihe Hospital, Hubei University of Medicine, Shiyan, China; ^3^ Department of General Surgery, Affiliated Haikou Hospital of Xiangya Medical College, Central South Univesity, Haikou, China

**Keywords:** breast cancer, cuprotosis, tumor microenvironments, prognosis, CRG

## Abstract

Breast cancer (BC) is now the most frequent and lethal cancer among women. Cuproptosis is a newly identified programmed cell death process that has been connected to tumor therapeutic sensitivity, patient outcomes, and the genesis of cancer. Cuproptosis-related genes (CRGs) are involved in breast cancer, although their roles and potential mechanisms are still unclear. First, we examined the effect of gene mutations and copy number changes on overall survival in 1168 breast cancer samples. Breast cancer patients were split into two molecular categories as determined by the variation in CRG based on clinicopathological traits, overall survival, and cell-infiltrating traits in tumor microenvironments. In addition, we created and validated a CRG score to calculate breast cancer patients' OS. Finally, we created a comprehensive nomogram for the clinical use of the CRG score. Patients whose CRG scores were low showed increased odds of developing OS, a larger mutation load, and immunological activation than those with high CRG scores. The CRG score, the cancer stem cell index, and the responsiveness to chemotherapy or targeted therapies were also shown to be statistically significantly correlated. Our thorough examination of CRGs in breast cancer patients demonstrated that they may be useful predictors of prognosis, clinical characteristics, and tumor microenvironment. These findings provide fresh insight into CRGs in breast cancer and might inspire brand-new approaches to both diagnosing and treating patients there.

## Introduction

There is a rapidly increasing incidence rate of breast cancer in women, which ranks first in terms of incidence and second in terms of mortality. The latest epidemiological statistics indicate that breast cancer accounts for approximately 30% of all new tumors in women (1, 2). With the continuous development of new targets and drugs for the treatment of breast cancer and the success of clinical trials involving new treatment protocols, the treatment and prognosis of breast cancer have advanced greatly (3–5). However, patients with advanced or high-risk conditions continue to have poor treatment outcomes and prognoses (6, 7). Early detection and rapid treatment would be very beneficial for patients with breast cancer, as they would increase their prognosis (8). In order to detect, diagnose, and treat breast cancer early, it is necessary to identify markers of the disease that are clinically very sensitive. Additionally, it is important to create more potent prognostic models.

Cuproptosis, a recently identified kind of programmed cell death, initiates an uncommon method of cell death, that is essential for several biological functions, such as mitochondrial metabolism (9). According to many studies, high copper levels in the blood and tissues of cancer patients may be a sign of a bad prognosis (10, 11). As a catalytic cofactor or structural component for cuproenzymes, copper is an essential metal ion in the majority of aerobic organisms and participates in a number of crucial biological processes (12). Tetrathiomolybdate, a copper ionophores and copper chelators used in anticancer therapy, has been linked to enhanced survival in advanced breast cancer (13–15). Previous studies have demonstrated that the serum copper level can potentially predict the prognosis of patients with BC (16). The discovery of many cuproptosis-related genes may provide fresh perspectives on treatment approaches and the prognosis of breast cancer patients.

Recent studies have indicated that cuproptosis may play a role in the occurrence, development and prognosis of a wide variety of cancers, suggesting that it could be used as a potential biological target in the diagnosis or treatment of these diseases (17–19). Until now, there have been no studies examining the role of cuproptosis in breast cancer and its tumor microenvironment; therefore, our study is the first to investigate the relationship between cuproptosis and breast cancer and its microenvironment. Using the algorithms CIBERSORT and ESTIMATE, the expression landscape of CRGs has been rigorously assessed and detailed immunological profiles have been produced. First, based on the levels of CRGs expression, we divided 1168 patients with breast cancer into two groups based on their molecular characteristics. The patients were divided into four gene subtypes based on the differentially expressed genes found for the two subtypes of cuproptosis. In the end, we created the CRG score method to forecast patients' outcomes by successfully predicting their overall survival from breast cancer. In conclusion, this study revealed that cuproptosis may serve as a new target for the diagnosis and (or) treatment of breast cancer, and that it thus provides a new research direction and/or idea and/or idea for the diagnosis and (or) treatment of breast cancer. 

## Materials and methods

### Collections of data

Based on data from The Cancer Genome Atlas (https://portal.gdc.cancer.gov/), information on RNA-sequencing raw data of 1110 cancerous breast samples as well as 112 normal human breast samples that included therapeutically information, somatic mutation data and CNV data files, was obtained. It was necessary to download processed gene expression datasets, clinical samples collected from breast cancer patients (n=58), as well as normal breast tissue (n=4) from the Gene Expression Omnibus profile database (https://www.ncbi.nlm.nih.gov/geo/) (ID: GSE61304). These raw data were first standardized to fragments per kilobase million expression levels prior to comparison and figuring out the expression of CRGs. After that, CRG expression was determined using the limma program (20). We integrated the data once the data cleaning procedure was finished to get them ready for analysis. The study that followed did not include patients for whom there was inadequate data on their survival.

### Analysis of CRGs using consensus clustering

19 CRGs made up the signature that we were able to collect from earlier publications (9, 21–25), the list of genes is in **Table S1**. We were able to classify individuals into discrete molecular clusters based on their CRG expression using the ConsensusClusterPlus R program (26). Through the use of unsupervised clustering, this was done. The clinical usefulness of CRGs in breast cancer was investigated using the Kaplan-Meier approach in a Kaplan-Meier study. We used the survival and survminer packages in R to examine the curves of survival as well as display the results. After that, the ggplot2 software was used to do a principal component analysis. The two subtypes' biological processes were maintained by using the Gene Set Variation Analysis tool (27). Malignant Tumour tissues employing expression  (28) and CIBERSORT (29) were also utilized to represent the percentage of immune and stromal cells in patients with breast cancer. The extent to which each immune cell within each sample carried an enrichment score was also assessed using an analysis of gene set enrichment on a single sample (30).

### Correlations between the subtypes and clinical features, and functional annotations

We associated the two cuproptosis-related subtypes with the primary clinical and pathological parameters of breast cancer patients, including their age, T phase, and N phase, as well as their prognosis, as part of our inquiry into the possible clinical functions of the two cuproptosis-related subtypes. Additionally, Kaplan-Meier survival analysis technique was utilized to look at differences in overall survival that were verified amongst the various subtypes. We discovered the differentially expressed genes between the cuproptosis-related subgroup using the limma R program. These genes required to possess an adjusted *p*-value < 0.05 and a fold change > 1.5. To clarify the pathways that were considerably enriched, gene ontology enrichment analysis and Kyoto Encyclopedia of Genes and Genomes pathway enrichment analysis were also conducted. To further explore the hidden roles among the DEGs, the data were displayed using the ClusterProfiler program (31).

### Creating and confirming the predictive CRG score

By computing the overall value of risk, the value of CRG was established in order to identify the cuproptosis patterns in specific patients. For a more thorough analysis, we utilized unsupervised consensus clustering to separate the breast cancer patients into four different subtype groups (cuproptosis-related gene subtype A-D). The train sets were then utilized to generate a CRG score for prognosis. A percentage of 1:1 was applied to all patient datasets in order to divide them into train and test sets. The glmnet package in R was used to perform least-squares regressions and selection operator regressions in order to minimize the possibility of overfitting the model (32). For the purposes of predicting the OS of the patients in the training set, a multivariate Cox regression with proportional hazards analysis was also utilized. Both the train set and the test set were split into groups of high-risk and low-risk based on their risk ratings. In each set, Kaplan-Meier analyses of survival and ROC curves were conducted.

### Clinical correlations and CRG-related prognostic model subgroup analyses

The relationships between the CRG score and the clinically significant parameters, including age, T and N stage, were examined using chi-square tests. On both the train and test sets, univariable and multivariable analyses were conducted to see if the CRG score was influenced by any other easily accessible clinicopathological characteristics. Age, tumor grade, T and N stage were also taken into account in subgroup studies to see whether the CRG score still had the same predictive value it did in our model earlier.

### Creation and verification of a nomogram

A nomogram was created using the rms program to predict overall survival based on clinically significant characteristics and the CRG score. Each clinicopathologically significant characteristic was given a score using the nomogram model, and the overall score was obtained by summing all the individual scores. By contrasting the area under the time-dependent ROC curves of survival rates after one, three, and five years, the nomogram's accuracy in predicting survival rates was also validated. Additionally, model calibration was performed to compare the predicted likelihood of survival outcomes across the 1-, 3-, and 5-year periods with the actual survival occurrences.

### Immune state and CSC index in high-risk and low-risk populations compared

To calculate the total number of tumor-infiltrating immune cells and subgroups of immune cells in each sample, we utilized the CIBERSORT method for comparison of 23 immune cells infiltrating the tumors between the high-risk and low-risk groups. This was done to assess how many immune cells altogether had invaded the tumor. The gene groups connected with 23 levels of immune cell infiltration were also found using the CRG score. Additionally, we looked at the connections between the CRG risk score and the cancer stem cell.

### Analysis of drug susceptibility to mutations

Depending on whether a sample was deemed high-risk or low-risk, its tumor mutation burden was assessed for each. Additionally, we used the maftools program to do a somatic variant analysis on patients with breast cancer in order to look at and analyse the somatic mutation data (33). Using the pRRophetic software, we calculated the semi-inhibitory concentrations (IC50) of frequently prescribed medications in breast cancer patients depending on their risk levels (34).

### Analyses of statistics

The R-based statistical analysis was conducted with a significance threshold of p 0.05 (version 4.0.2). 

## Results

### 19 CRGs in breast cancer: Expression, genetic variants, and prognostic values

**Figure 1:**
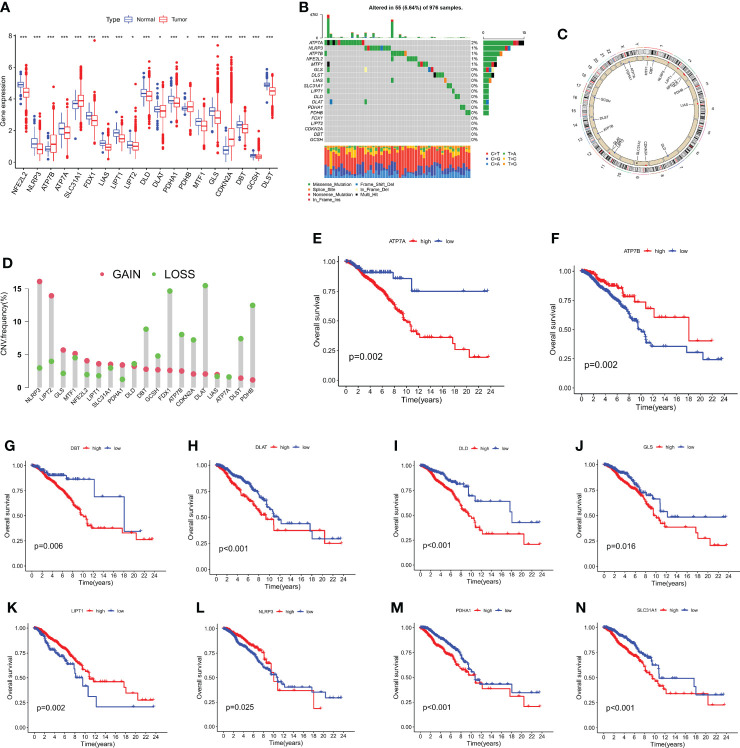
The analysis of 19 CRGs' expression and association in the TCGA cohort. **(A)** The expression of the 19 CRGs in BC tissues and healthy breast tissues (*p < 0.05; ***p < 0.001). **(B)** Data on the frequency of CRG mutations for 976 BC patients. **(C)** The sites of CNV variation in CRGs on the 23 chromosomes. **(D)** The distributions of CNV gain, loss, and non-CNV among CRGs. **(E–N)** The association between 10 CRGs and overall survival in British Columbia.

On the TCGA dataset, 1110 breast cancer patients' expression levels of 19 cuproptosis-related genes were examined, along with 112 normal human breast tissues (**Figure 1A**). In the meanwhile, gene mutation analyses revealed that 55 out of the 976 samples (5.64%) had CRGs mutations, with *ATP7A* having the greatest gene mutation rates (**Figure 1B**). The majority of CRGs were accumulated on copy number loss or deletion, according to an examination of copy number variations (**Figure 1C**), and all 19 CRGs had frequent copy number alterations (**Figure 1D**). Additionally, it was discovered *via* research of the impact of gene expression patterns on overall survival in breast cancer that those expressing high levels of *ATP7A, DBT, DLAT, DLD, GLS, PDHA1*, and *SLC31A1* had a bad prognosis. A higher level of* ATP7B, LIPT1*, and* NLRP3* expression is linked to improved OS (**Figure 1E–N**, and **Table S2**). The findings suggested that CNV alterations could modify the way CRGs are expressed. Additionally, a relationship between CRG expression levels and breast cancer prognostic variables was discovered, pointing to a potential involvement for CRGs in breast cancer. The biomarkers might be used as therapeutic targets or prediction biomarkers for breast cancer.

### Subtypes of cuproptosis are identified in breast cancer

**Figure 2:**
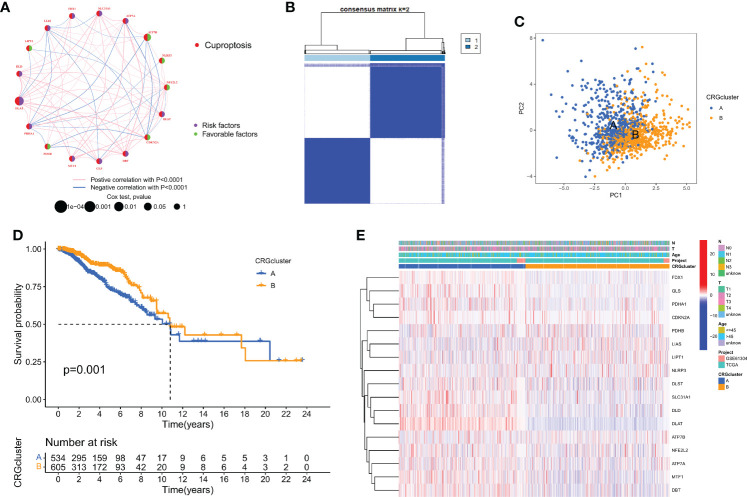
Biological and clinicopathological characteristics of CRG subtypes. **(A)** The interactions between CRGs in BC (the red and blue strings denote positive and negative correlation, respectively; the intensity of the correlation is indicated by the color shades). **(B)** The consensus matrix's heatmap of two clusters (κ = 2). **(C)** A considerable transcriptome divergence between the two subtypes is seen by PCA analysis. **(D)** Subtype-specific Kaplan-Meier OS curves. **(E)** CRG expression levels and clinicopathological traits vary across subtypes.

The correlation network picture showed the 19 CRGs' strong association with one another (**Figure 2A**). The cohort was subdivided into two groups, group A (n = 534) and group B (n = 605), based on a consensus cluster analysis of the 1168 breast cancer samples, which showed that a cluster of κ = 2 had the largest intragroup and lowest intergroup differences (**Figure 2B**, and **Figure S1**). Differences in the transcription patterns of the two subtypes of cuproptosis were found using PCA (**Figure 2C**). Subtype B has a better prognosis than subtype A, according to Kaplan-Meier survival calculations (*p* = 0.001; **Figure 2D**). A heatmap was created as a consequence of the relationship between features of clinical significance and patterns of CRG expression (**Figure 2E**). The bulk of CRGs expressed themselves more strongly in subtype A, whereas late-phase breast cancer was represented in subtype B.

### Analyses of TME infiltration and functional enrichment in distinct subtypes

**Figure 3:**
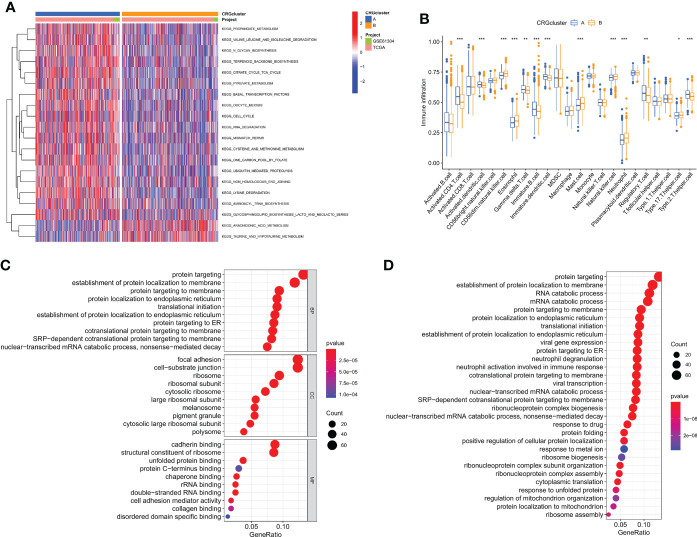
Cuproptosis subtypes linked to TME invasion. **(A)** GSVA of two cuproptosis subtype-related cellular pathways (Red means activated and blue means inhibited). **(B)** Correlations between immune cell infiltration levels in the two subtypes associated with cuproptosis. **(C, D)** DEG enrichment studies across two cuproptosis-related subgroups using GO and KEGG. *p < 0.05, **p < 0.01, ***p < 0.001.

We used gene set variation analysis enrichment analysis to look at the two subtypes' possible effects on biological behavior (**Figure 3A**). Compared to subtype B, subtype A had an enrichment in the pathways linked to immunological activation. According to a GSVA enrichment study, subtype A is considerably enriched in metabolic-activated pathways, such as the folate utilization of one carbon pool, lysine degradation, the citrate cycle, RNA metabolism, arachidonic acid metabolism, and N-glycan biosynthesis. In each breast cancer sample, we used the CIBERSORT method to assess the associations between two subtypes as well as the 23 other subtypes of immune cells in order to learn more about how CRGs work in the tumor microenvironment. According to our research, there are significant variations between the two subtypes in the quantity of immune cells that infiltrate (**Figure 3B**). As compared to subtype B, CD4 T cells, type 2 T helper cells, regulation T cells, gamma delta T cells, immature dendritic cells, and immature B cells were found to be more prevalent in subtype A. Subtype A, on the other hand, exhibited considerably reduced levels of neutrophil, eosinophil, mast cell, and CD56 dim natural killer cell infiltration. Then, we did a functional enrichment analysis to look into the two cuproptosis subtypes' possible biological roles after using the limma algorithm to identify 591 DEGs linked to them (**Figure S2** and **Table S3**). It was discovered that CRGs were mainly engaged in membrane protein targeting, membrane protein localization establishment, and pathway analysis using GO and KEGG (**Figures 3C, D**, and **Figure S3**).

### Gene subtypes are identified using DEGs

**Figure 4:**
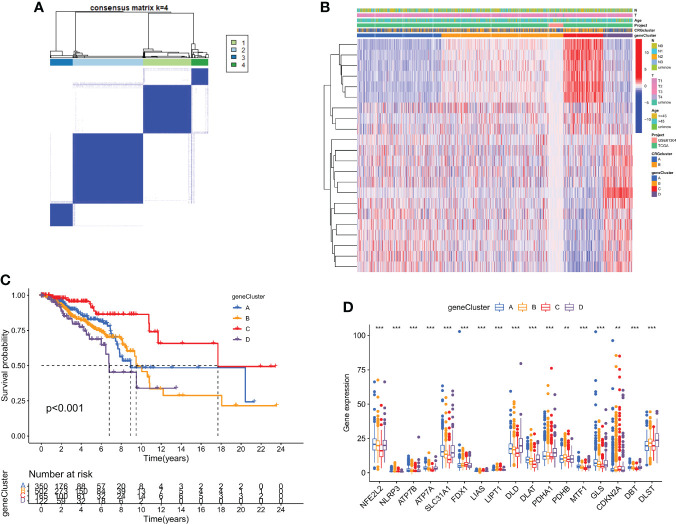
DEGs are used to identify gene subtypes. **(A)** Heatmap of the consensus matrix defining four clusters ( κ = 4). **(B)** Differences in clinicopathologic characteristics among the four gene subtypes. **(C)** The four gene subtypes' Kaplan-Meier OS curves. **(D)** Variations in the expression of ten CRGs across four gene subtypes. **p < 0.01, ***p < 0.001.

Using a consensus clustering technique, 1139 breast cancer patients were categorized into four molecular genetic categories based on prognostic genes. Subtypes A (n = 350), B (n = 502), C (n = 165), and D (n = 122) were found when κ = 4 indicated that the breast cancer instances may be separated into four subclasses (**Figure 4A** and **Figure S4**). Additionally, the relationship between the clinical traits of breast cancer patients and the gene subtypes was investigated (**Figure 4B**). The genetic subtype D patients had the lowest OS, while patients with genetic cluster C had the greatest OS, according to Kaplan-Meier curves (*p* < 0.001; **Figure 4C**). The four cuproptosis gene subtypes' expression of CRGs varied greatly, as expected by the cuproptosis patterns (**Figure 4D**).

### Creating and confirming the predictive CRG score

**Figure 5:**
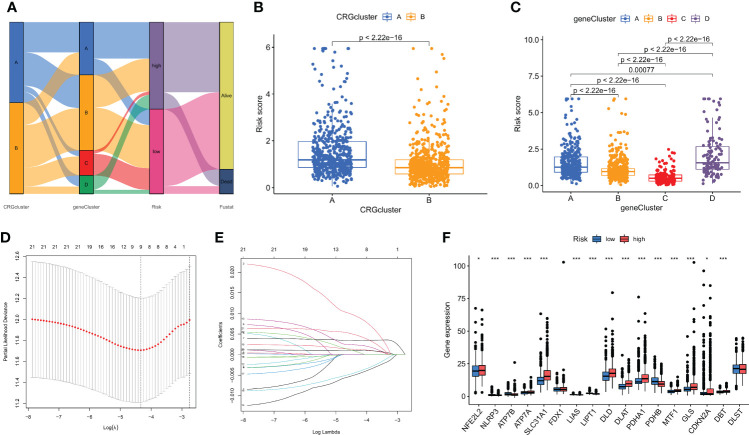
The CRG score was created in the TCGA and GSE61304 cohorts. **(A)** The subtype distributions among groups, CRG scores and survival outcomes. **(B)** Variations in CRG scores among cuproptosis subtypes. **(C)** Variations in PRG scores among different gene subtypes. **(D)** CRG regression using LASSO. **(E)** Cross-validation of LASSO regression parameter selection. **(F)** CRG score differences in ten CRGs. *p < 0.05, ***p < 0.001.

Based on DEGs related to subtypes, a LASSO-Cox regression model was developed to provide a predictive CRG score for each patient. **Figure 5A** illustrates the proportion of patients among the two CRG score groups, the two cuproptosis subtypes, and the four gene subtypes. There was a statistically significant variation in CRG scores across cuproptosis subtypes. Subtype B had a much higher CRG score than subtype A. **Figure 5B** displays the risk score distributions for the two CRG subtypes. The highest CRG scores were for subtype D, while the lowest were for subtype C (**Figure 5C**).

**Figure 6:**
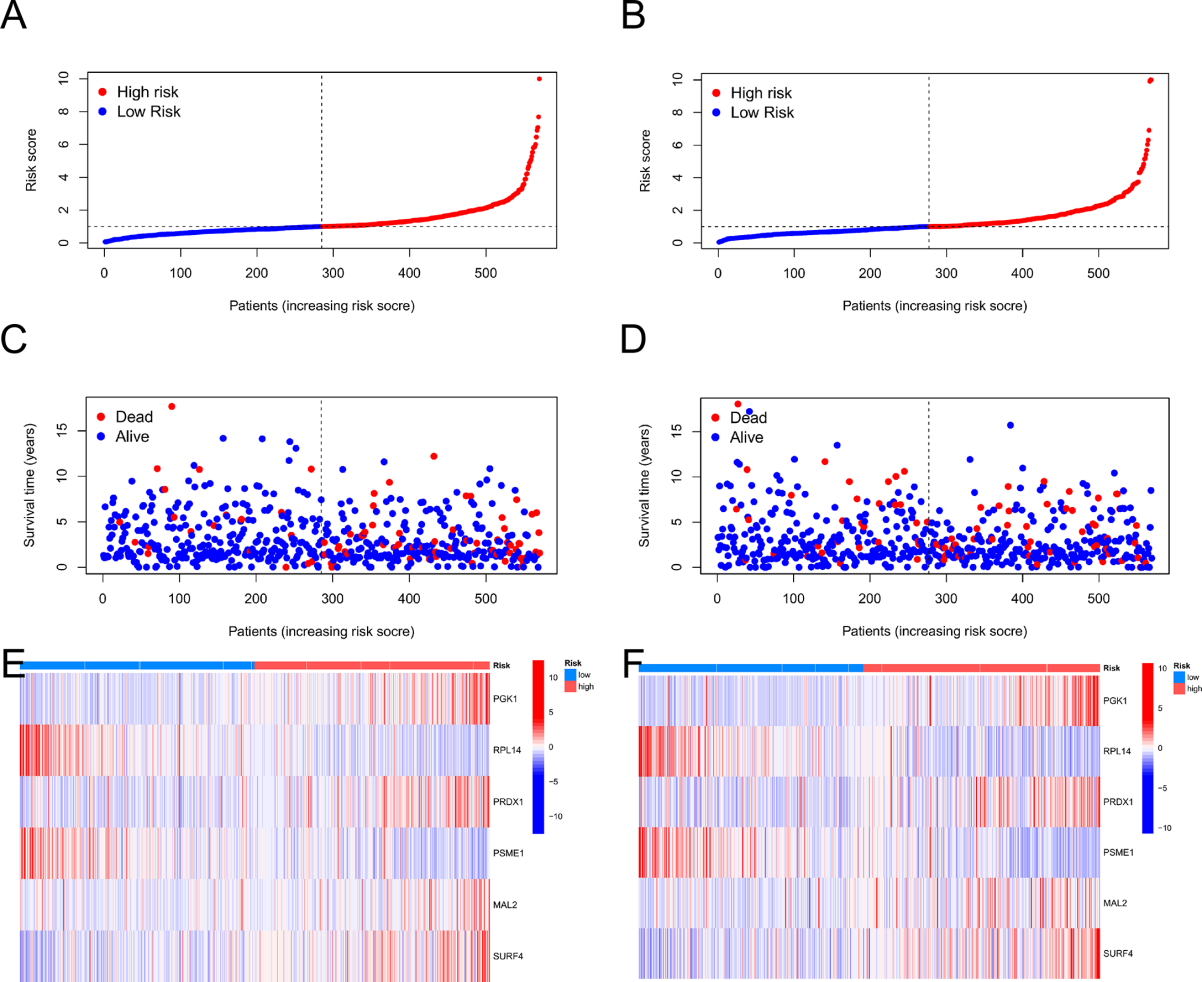
The patient survival status and CRG score distribution vary between the train and test sets. **(A, C, E)** The patient survival status and CRG score distribution in the train set. **(B, D, F)** The patient survival status and CRG score distribution in the test set.

Then, using R's caret package, patients were randomly assigned to training groups (n = 570) as well as testing groups (n = 569) at a ratio of 1:1 (**Tables S4, S5**). Using LASSO and multivariate Cox analysis, 22 OS-related genes were selected using the least partial likelihood deviation from 591 cuproptosis subtype-related prognostic DEGs (**Figures 5D, E**, and **Table S6**). Based on a Cox regression analysis involving several variables, Akaike information criteria value of 22 OS-associated genes was utilized to identify six genes (*PGK1, RPL14, PRDX1, PSME1, MAL2*, and *SURF4*) (**Table S7**). These findings led to the following formula being chosen as the risk score formula: The risk score is calculated as follows: (0.00375* PGK1 expression) + (-0.00930*RPL14 expression) + (0.00278*PRDX1 expression) + (-0.00668*PSME1 expression) + (0.00147*MAL2 expression) + (0.00672*SURF4 expression). 13 out of 17 hallmark genes showed a significant variation in their expression of genes between high-risk individuals and low-risk individuals (**Figure 5F**). Based on their risk ratings, each theme was split into high- and low-risk patient groups, and the median scores were calculated for the training and test sets. According to their values of risk, patients were split into two groups: those at low risk and those at high risk (**Figure 6**). In terms of survival rates and circumstances, there were significant differences among the two groups based on Kaplan-Meier curves. Patient survival rates and the distribution of CRG scores were analyzed independently for the train and test sets.

### Creating a nomogram to forecast survival

**Figure 7:**
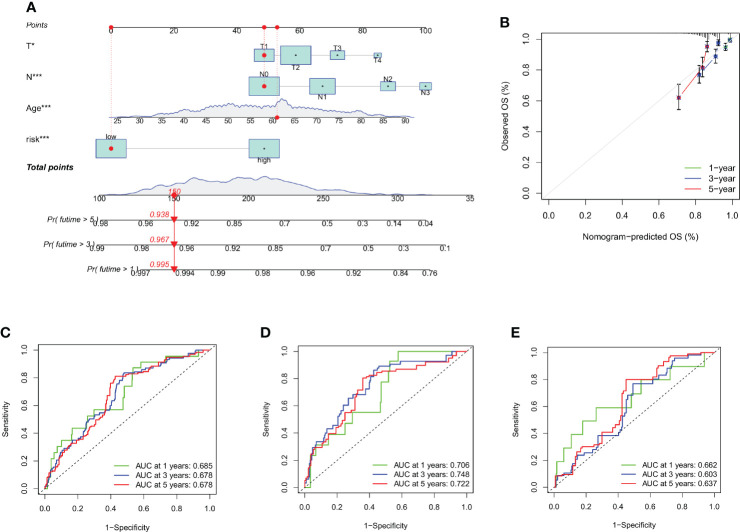
Creating and evaluating a nomogram. **(A)** The nomogram used to calculate the survival rates of 1-, 3-, and 5-years for patients with BC. **(B)** Calibration curve for nomograms. **(C–E)** ROC curves for the train set and test set, respectively, for forecasting 1-, 3-, and 5-year OS in the cohorts. *p < 0.05, ***p < 0.001.

Using the data gathered, we created a nomogram using the rms program to forecast the life expectancy of breast cancer patients at the lifetime of 1, 3, and 5 (**Figure 7A**, and **Table S8**). Each patient's total point values were determined based on prognostic characteristics such as their age, level of risk (low risk was indicated by a "low CRG score" and high risk was indicated by a "high CRG score"), as well as the T and N stage of their ailment. The harshness of the prognosis is directly correlated with the patient's overall score. The calibration plots showed that the nomogram performed better than an ideal model would have (**Figure 7B**). Additionally, ROC analysis indicated that the nomogram performed very well in terms of prediction (**Figures 7C–E**).

### Relationship of TME and Mutation burden with CRG score

**Figure 8:**
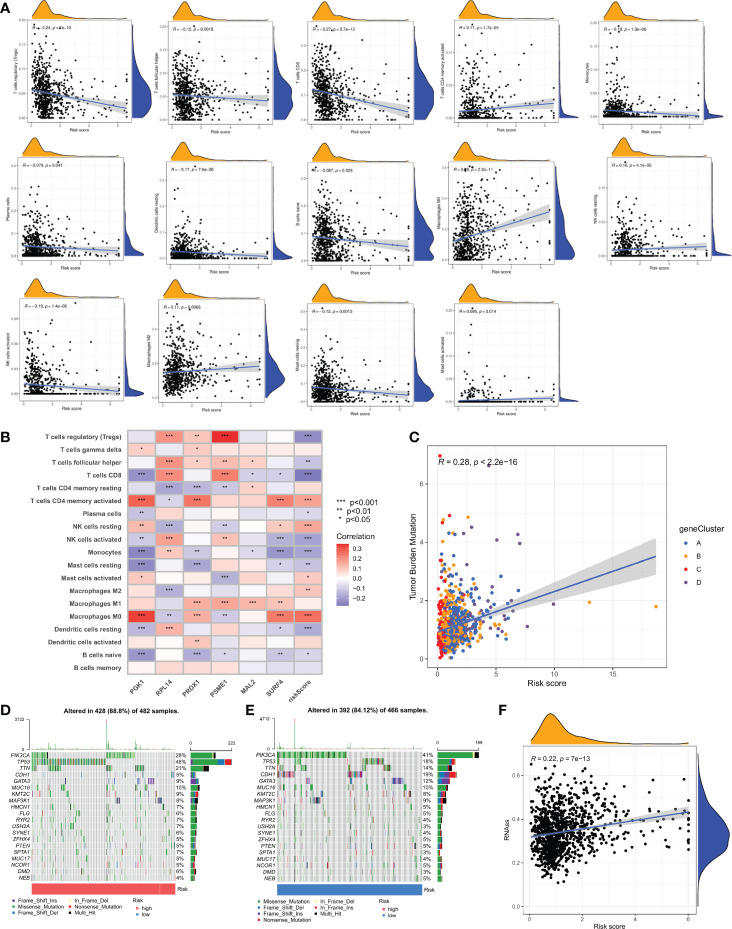
Comprehensive analysis of the CRG scores in BC. **(A)** Correlations between immune cell types and CRG score. **(B)** The six genes from the proposed model are correlated with the number of immune cells. **(C)** CRG score and TMB spearman correlation analysis. **(D, E)** The somatic mutation features waterfall plot determined by high and low CRG scores. One patient was represented by each column. The correct number represented each gene's frequency of mutation, and the upper barplot displayed TMB. The proportion of each variant type was displayed in the right barplot. **(F)** Associates between the CSC index and the CRG score.

The CIBERSORT algorithm was used to assess the relationship between the CRG score (**Figure S5**) and the number of immune cells. However, the CRG score was negatively correlated with naive B cells, resting dendritic cells, resting mast cells, monocytes, activated NK cells, plasma cells, CD8 + T cells, and follicular helper T cells. A correlation was found between the CRG score and activated memory CD4 + T cells, M0 macrophages, M2 macrophages, activated mast cells, and resting NK cells (**Figure 8A**). Additionally, our research looked at the association between six genes and the amount of immune cells. According to our study, the six genes affect the bulk of immune cells (**Figure 8B**, and **Table S9**).

The TMB study revealed a significant association between anticipated TMB level and cuproptosis gene subtypes (R = 0.28, *P* < 0.001; **Figure 8C**). To give further support, we looked at the variations in somatic mutation distribution across the cohort's two CRG score groups. The top 10 most changed genes in each of the two groups were *PIK3CA, TP53, TTN, CDH1, GATA3, MUC16, MAP3K1, HMCN1*, and *FLG*. The most often mutated genes in patients with a high CRG score are *TP53* (46%) and *PIK3CA* (28%), while *PIK3CA* (41%) is the most frequently mutated gene in the low-risk category (**Figures 8D, E**).

### Drug susceptibility testing and CSC index

**Figure 9:**
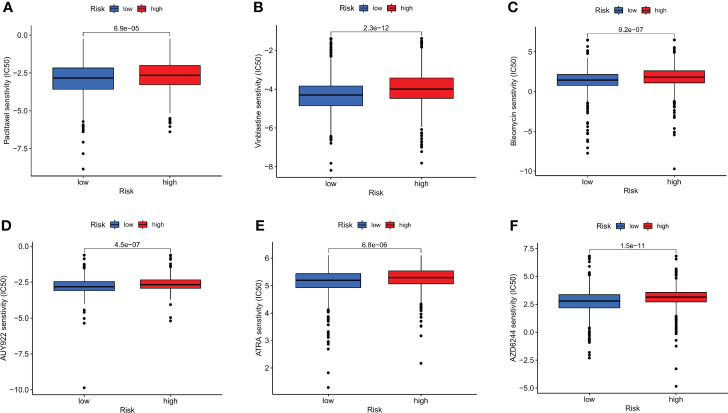
Relationships between the CRG score and susceptibility to chemotherapy or targeted therapies for BC. **(A)** paclitaxel. **(B)** Vinblastine. **(C)** Bleomycin. **(D)** AUY922. **(E)** ATRA. **(F)** AZD6244.

Additionally, it was shown that there was a link between the CRG score and the CSC index that was positive (R = 0.22, *P* < 0.001), suggesting that cells from breast cancer with higher cell retention gene scores demonstrated more stem cell features and less differentiation (**Figure 8F**). Sensitivity analysis was done on a few medications presently being used to treat breast cancer among the two groups. For patients with high CRG scores, it was found that the IC50 values of drugs including paclitaxel, vinblastine, bleomycin, AUY922, ATRA, and AZD6244, among others, were considerably higher. It is evident from these results that CRGs are essential for the sensitivity of drugs (**Figures 9A–F**).

## Discussion

Breast cancer is a potentially deadly illness that places a heavy burden on people worldwide (1–3). It is vital to first identify people who are more likely to get the illness, and then find measures to lower that risk, in order to decrease the prevalence of breast cancer (35, 36). If more study is done on innovative processes and treatments, a higher proportion of patients will be cured (37). We are aware of very little research that have looked at potential connections between CRGs and breast cancer in the past. Our research showed that when compared to normal tissues, breast cancer tissues expressed the majority of CRGs at varying levels. Furthermore, cuproptosis may have prognostic or predictive value in patients with breast cancer in accordance with the level of expression of these genes in these individuals.

Several studies have connected copper to human cancer tumor cell development, proliferation, and carcinogenesis (21–25, 38–41). However, additional investigation is needed to pinpoint the specific pathways, which include tumor initiator cells, growth, and metastatic spread, and to demonstrate causal linkages between copper and human cancer. It has not yet been completely determined how important these effects and immune infiltration characteristics caused by several CRGs are. Our research showed that both genetic and transcriptional alterations occurred in CRGs in breast cancer. On the basis of CRGs, our study identified two distinct molecular subtypes. Patients with subtype A had more severe clinical characteristics and shorter OS compared to those with subtype B. Individuals with high expression of* ATP7A, DBT, DLAT, DLD, GLS, PDHA1*, and *SLC31A1 *have a bad prognosis, while those with high expression of *ATP7B, LIPT1* and *NLRP3* have a favorable prognosis. The effect of gene expression patterns on overall survival in breast cancer was also studied. Additionally, we contrasted variations in the traits and immunologically-related biochemical pathways of the two TME subtypes. As a result of the activation of CD4 T cells, eosinophils, gamma delta T cells, regulatory T cells, mast cells, active dendritic cells, neutrophils, type 2 T helper cells, CD56 dim natural killer cells, immature dendritic cells, and immature B cells, the immunological activation of the breast cancer subtypes was also substantial. Then, four gene subtypes were determined using the DEGs between the two cuproptosis subtypes. In addition, we developed the prognostic CRG score and demonstrated its tendency for prediction. In comparison with patients with low-risk CRG values and those with high-risk CRG values, there were significant variations in overall survival, clinical traits, mutations, TME, CSC index, and medication resistance. Finally, to improve performance and make the CRG score simpler to use, we developed a nomogram that was derived from patient characteristics and the CRG score. The prognostic model may encourage beneficial understandings of the molecular basis of breast cancer as well as fresh approaches to cancer treatment.

Recent studies have revealed that cuproptosis plays an important role in human tumor. Bian Z, et al. examined the genetic alterations of cuproptosis-associated genes in clear cell renal cell carcinoma (17). Han J, et al. investigated the prognostic role of cuproptosis-related long non-coding RNAs in soft tissue sarcoma and its correlation with the tumor microenvironment (18). According to Zhang Z, et al., cuproptosis-related genes are useful for clinical prediction of prognosis and treatment guidance in hepatocellular carcinoma (42). The relationship between cuproptosis and breast cancer and its microenvironment has not previously been studied; thus, our study serves as the first to examine this relationship. Our study shows that copper death-related genes are differentially expressed in breast cancer and are associated with OS in patients with breast cancer, which may assist in predicting the prognosis for breast cancer patients. Copper has been shown to play an important role in tumor development and can be used to predict the prognosis and treatment of tumors (13–16). Patients with different cuproptosis-related0 subtypes exhibit different characteristics and tumor microenvironment, and patients in high and low risk groups differ in their sensitivity to treatment. Consequently, we speculate that different treatment approaches for different subtypes of patients may produce better outcomes, however, this hypothesis requires further validation *in vivo* and *in vitro*.

As is well known, the tumor microenvironment is made up of both the tumor cells and the cells that surround them, such as lymphocytes, tumor infiltrating immune cells, and the tumor vasculature (41–43). There is strong evidence to back up the idea that TME is essential for tumor formation, progression, and therapy resistance (44–46). In the present investigation, we found that the TME features as well as the abundances of 23 TIICs were substantially varied across the two distinct molecular subtypes and the various CRG scores. This result suggests that CRGs are essential to the growth of breast cancer. When TIICs are found in tumor tissues, breast cancer patients have a better prognosis. Activated CD4 T cells, type 2 T helper cells, gamma delta T cells, regulatory T cells, immature dendritic cells, immature B cells, and activated dendritic cells were more prevalent in Type A subtypes than Type B subtypes, according to the findings of our study. It was discovered that subtype B had much reduced numbers of eosinophils, mast cells, neutrophils, and CD56 dim natural killer cells infiltration. Given the success of immunotherapy in breast cancer, research on the tumor microenvironment and immune cell infiltration can help discover new directions and mechanisms of immunotherapy for breast cancer.

This study has the following contributions. First of all, this research is the first of its kind to identify subtypes associated with cuproptosis and create a predictive model based on CRGs in breast cancer. Because cuproptosis differs from other recognized methods of cell death, it may provide new therapeutic possibilities for treating cancer (47, 48). Second, a variety of different techniques and databases were employed. As a means of improving the reliability of our findings, we also defined subtypes associated with cuproptosis and created a predictive model for use in screening and testing processes.

There are several restrictions on our research. First, the studies solely used data from public sources; additional validations using more accurate clinical data are required. Additionally, it was not feasible to analyze data for several critical clinical factors (surgery, chemoradiotherapy, and radiation therapy), which would have had an impact on the immune response and drug susceptibility prognosis. Since the prognostic signature was created and verified using data from publicly available sources, more experimental investigations as well as extensive prospective studies are required to corroborate our results. 

## Conclusion

In this study, we systematically analyzed the role of cuproptosis-related genes in breast cancer prognosis and correlation with tumor microenvironment and clinical features, and constructed a better prognostic prediction model. We also explored the effectiveness of CRGs as biomarkers of response to therapy. In conclusion, our study reveals the clinical importance of CRGs, which provides a valuable basis for further studies on the diagnosis or personalized treatment of breast cancer patients.

## Data availability statement

The datasets presented in this study can be found in online repositories. The names of the repository/repositories and accession number(s) can be found in the article/**Supplementary Material**.

## Author contributions

ZL, GW, and JZ contributed to the conception and design of this study. XW, HZ, and QW collected and analyzed the data. YS, JX, and ZL drafted the original manuscript. MW and GW polished and revised the manuscript. This manuscript has been read and approved by all authors.

## Acknowledgments

Data contributions from the databases of the TCGA and GEO are appreciated by the authors.

## Conflict of interest

The authors declare that the research was conducted in the absence of any commercial or financial relationships that could be construed as a potential conflict of interest.

## Publisher’s note

All claims expressed in this article are solely those of the authors and do not necessarily represent those of their affiliated organizations, or those of the publisher, the editors and the reviewers. Any product that may be evaluated in this article, or claim that may be made by its manufacturer, is not guaranteed or endorsed by the publisher.

## Abbreviations

BC, Breast cancer; CRGs, cuproptosis-related genes; TCGA, the cancer genome atlas; DEGs, differentially expressed genes; GSVA, gene set variation analysis; GEO, the gene expression omnibus; TMB, tumor mutation burden; OS, overall survival; CSC, cancer stem cell; TME, tumor microenvironment; ROC, receiver operating characteristic; TIICs, tumor-infiltrating immune cells.
