# Supplementary material for: Identification of cuproptosis-related subtypes, characterization of tumor microenvironment infiltration, and development of a prognosis model in breast cancer
Source: Front Immunol. 2022 Aug 19;13:996836. doi: 10.3389/fimmu.2022.996836 (PMC9437337; doi:10.3389/fimmu.2022.996836)
Supplement: Supplementary file 1 [file DataSheet_1.docx]

**Supplementary Figures**


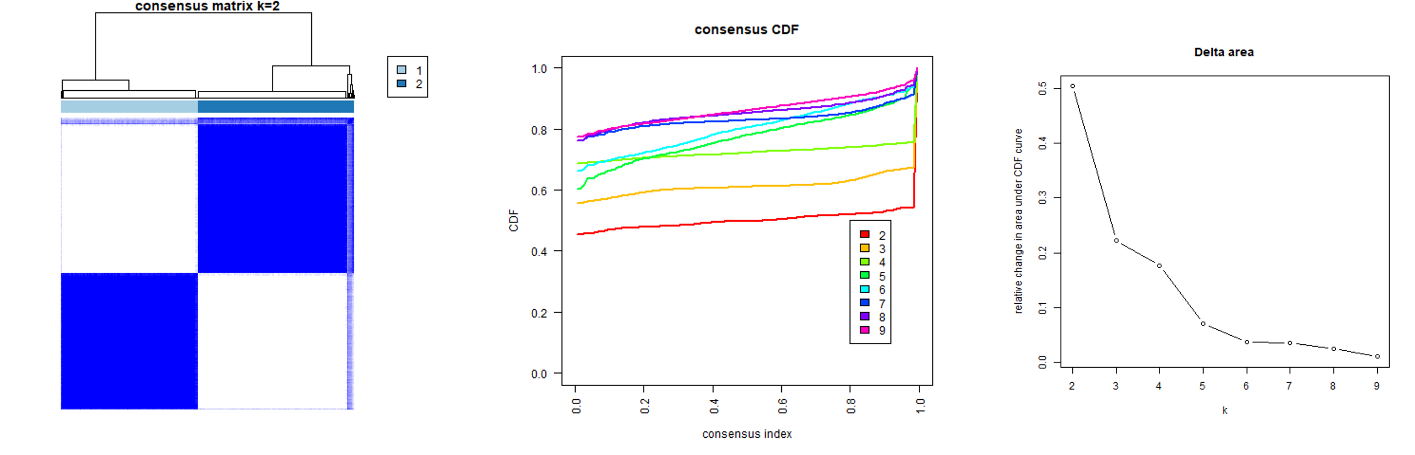


**Figure S1** Identification of cuproptosis-related subtypes in BC cohort.


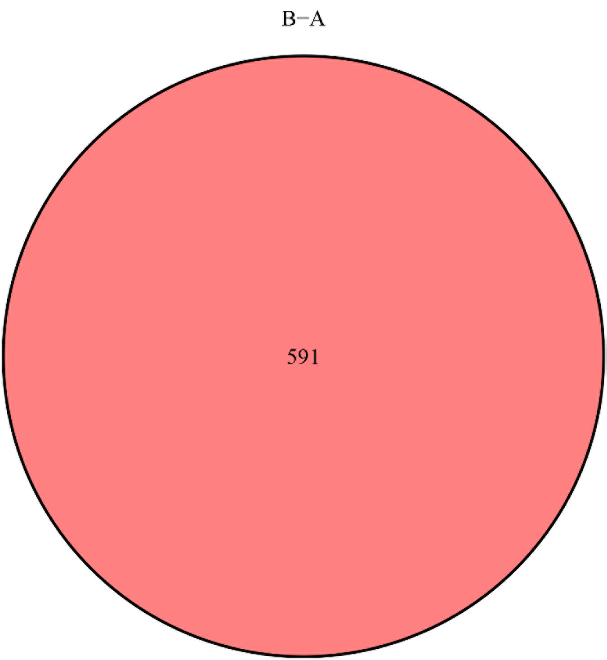


**Figure S2** VennDiagram of 591 DEGs.

**
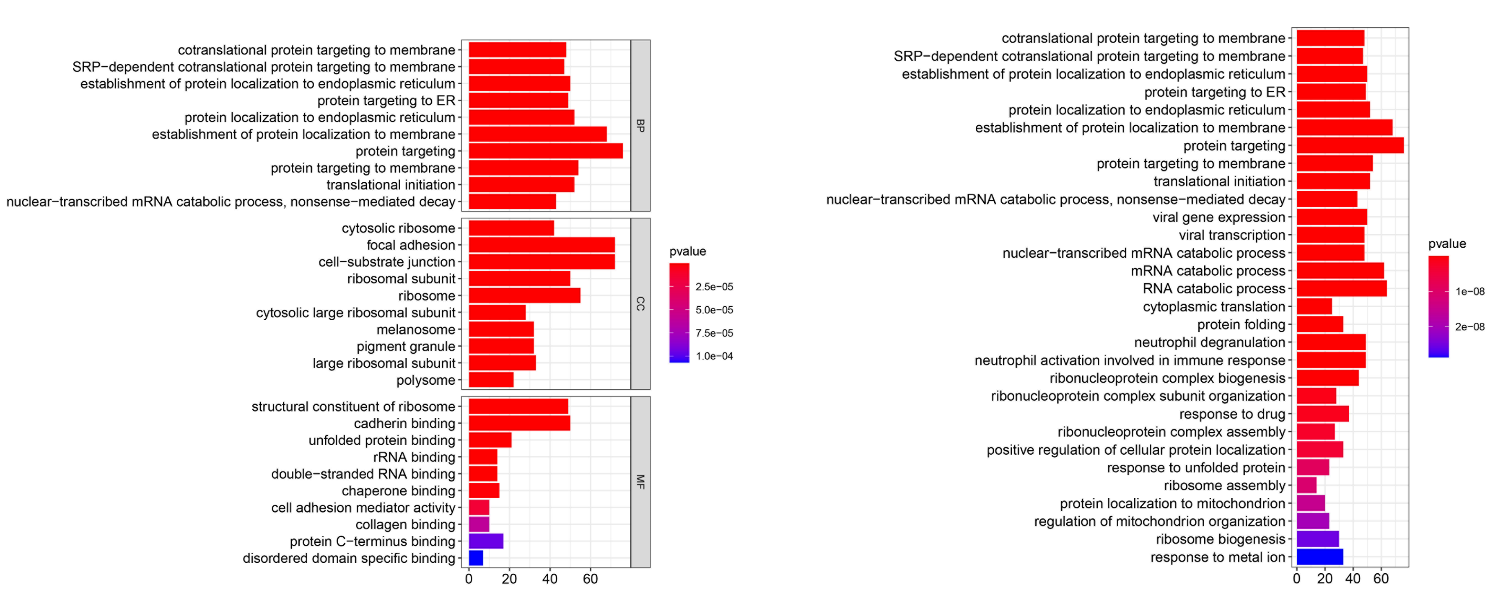
**

**Figure S3** Barplot of GO and KEGG analysis.

**
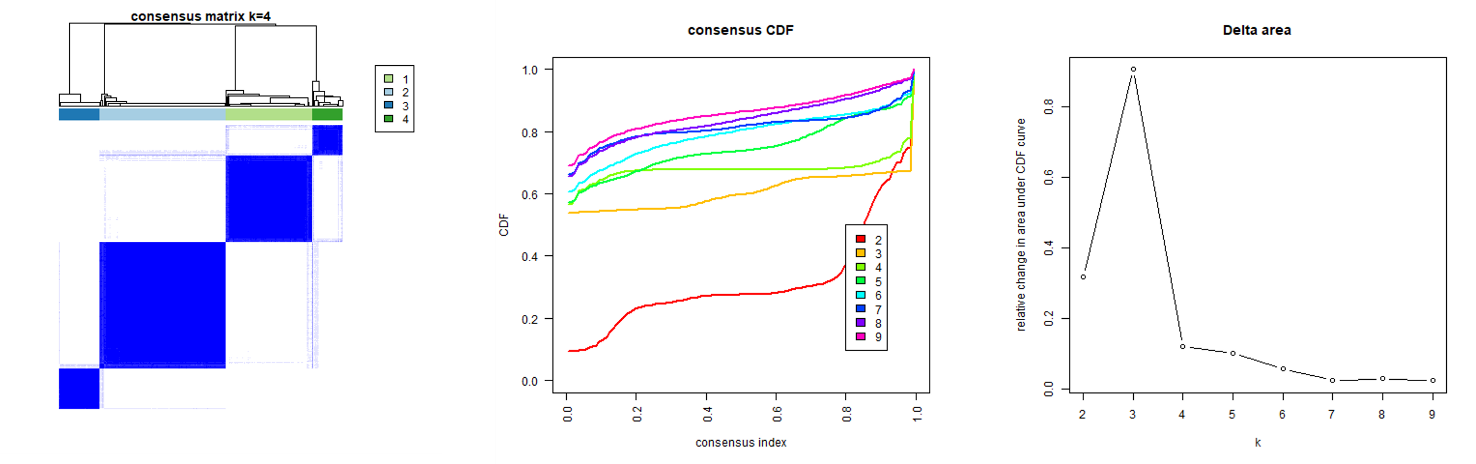
**

**Figure S4** Identification of cuproptosis-related gene subtypes in BC cohort.

**
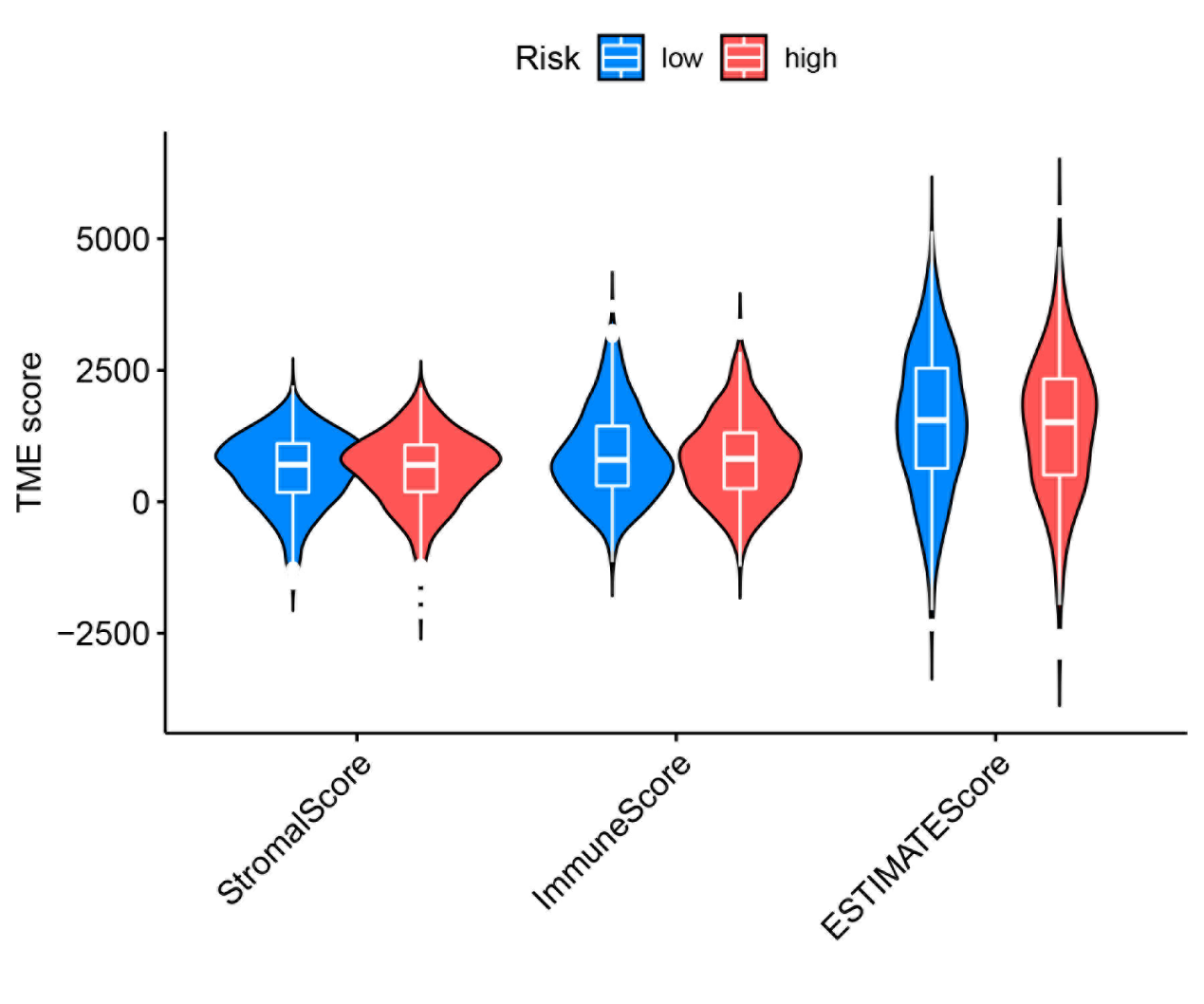
**

**Figure S5** TME score.
